# A Pre- and Co-Knockdown of RNAseT Enzyme, Eri-1, Enhances the Efficiency of RNAi Induced Gene Silencing in *Caenorhabditis elegans*

**DOI:** 10.1371/journal.pone.0087635

**Published:** 2014-01-24

**Authors:** Pooja Jadiya, Aamir Nazir

**Affiliations:** 1 Academy of Scientific and Innovative Research (AcSIR), Anusandhan Bhawan, New Delhi, India; 2 Laboratory of Functional Genomics and Molecular Toxicology, Division of Toxicology, CSIR-Central Drug Research Institute, Lucknow, India; French National Center for Scientific Research - Institut de biologie moléculaire et cellulaire, France

## Abstract

**Background:**

The approach of RNAi mediated gene knockdown, employing exogenous dsRNA, is being beneficially exploited in various fields of functional genomics. The immense utility of the approach came to fore from studies with model system *C. elegans*, but quickly became applicable with varied research models ranging from *in vitro* to various *in vivo* systems. Previously, there have been reports on the refractoriness of the neuronal cells to RNAi mediated gene silencing following which several modulators like *eri-1* and *lin-15* were described in *C. elegans* which, when present, would negatively impact the gene knockdown.

**Methodology/Principal Findings:**

Taking a clue from these findings, we went on to screen hypothesis-driven- methodologies towards exploring the efficiency in the process of RNAi under various experimental conditions, wherein these genes would be knocked down preceding to, or concurrently with, the knocking down of a gene of interest. For determining the efficiency of gene knockdown, we chose to study visually stark phenotypes of uncoordinated movement, dumpy body morphology and blistered cuticle obtained by knocking down of genes *unc-73, dpy-9* and *bli-3* respectively, employing the RNAi-by-feeding protocol in model system *C. elegans*.

**Conclusions/Significance:**

Our studies led to a very interesting outcome as the results reveal that amongst various methods tested, pre-incubation with *eri-1* dsRNA synthesizing bacteria followed by co-incubation with *eri-1* and gene-of-interest dsRNA synthesizing bacteria leads to the most efficient gene silencing as observed by the analysis of marker phenotypes. This provides an approach for effectively employing RNAi induced gene silencing while working with different genetic backgrounds including transgenic and mutant strains.

## Introduction

RNA interference (RNAi) is an immensely valuable tool in functional genomics studies for determining the function of specific gene. This phenomenon was first described by Andrew Fire and Craig Mello in 1998 in the nematode *Caenorhabditis elegans* [1]. This sequence specific gene silencing method is highly conserved among all eukaryotic organisms [2], [3] which is triggered by the introduction of double-stranded RNA (dsRNA) into the cytoplasm. This dsRNA is first cleaved by Dicer, a member of the RNase III family, into short interfering RNAs (siRNAs) of approx. 21–23 nucleotides in length which ‘label’ homologous mRNA for degradation. Thus, the expression of a particular gene is suppressed and gene specific loss of function, phenotypes and potent interference is rapidly produced.

In *C. elegans*, there are three methods for delivering dsRNA for RNAi, including microinjection [1], soaking [4] and feeding [5]. Amongst these, RNAi by feeding is the most widely employed in various studies including large scale genetic screens for functional genomics analyses. Previous studies have shown that effectiveness of RNAi varies depending on the cell type, organism or target sequence and RNAi works less efficiently in the nervous system of *C. elegans* [6], [7] and also the efficacy and sensitivity of RNAi using wild type *C. elegans* (N2 Bristol) is sometimes limited [8]. On the other hand, it has been revealed that the efficiency and robustness of high throughput RNAi screens could be improved by using *rrf-3* deletion mutant [6], [8]. Mutants of *rrf-3*, a putative RNA-directed RNA polymerase (RdRP), are generally more sensitive to RNAi than wild type worms. However, the major need to devise method for enhancement of RNAi efficiency in wild type and neuronal tissues comes in to play when the experiments need to be performed in non *rrf-3* background. Earlier it has been shown that enhanced RNAi (Eri) mutants increase the sensitivity of worms to dsRNA in most of the tissues including, nervous system with enhancement of RNAi phenotypes in large-scale screens [9]. Besides, recent studies have also revealed that *C. elegans* mutant strain *lin-35* is also more sensitive to RNAi [10], [11]. Whereas these mutant backgrounds provide with an environment for enhanced RNAi induced gene silencing; same is not available while working with other genetic backgrounds like specific mutants and transgenic strains which have been constructed in a wild type background. We endeavored to study different conditions of RNAi methodology towards devising a strategy that could exploit the effects of *eri-1* or *lin-35* knockdown so as to make it possible to have enhanced RNAi efficiency even while working with strains not harbouring these specific mutations. We, hence, selected loss of *eri-1* and *lin-35*, the two known RNAi enhancers to examine the efficiency of RNAi with some known phenotypic gene markers i.e. *unc-73*, *dpy-9* and *bli-3*, which when efficiently knocked down, would lead to visibly marked phenotypes of uncoordinated movement, dumpy body and blistered cuticle; the phenotypes that have already been reported and associated to these genes. [8].

## Materials and Methods

### *C. elegans* culture and maintenance

Maintenance and culture of *C. elegans* were carried out at 22°C using standard protocol as described [12], [13]. In brief, worms were grown on a lawn of *Escherichia coli* OP50 seeded Nutrient Growth medium (NGM) which was prepared by adding 50 mM Sodium chloride (Merck), 2.5 gL^−1^ Peptone (Sigma), 17 gL^−1^ Agar (Hi-media) in 975 ml double distilled water and autoclaved for 30 to 40 minutes at 15 lb/inch^2^. After the cooling of media to 50°C–60°C, cholesterol solution (Sigma) prepared in ethanol, Calcium chloride (Sigma), Magnesium Sulphate (Sigma) and Potassium dihydrogen phosphate (SRL) were added to a final concentration of 5 µgml^−1^, 1 mM, 1 mM and 25 mM respectively. On the day of initiation of treatment, gravid nematode populations were synchronized by hypochlorite bleaching for isolation of embryos so as to have a synchronous population of nematodes [14]. The isolated embryos were subsequently cultured on NGM plates with OP50 and analyzed after 48 hrs of treatment. In this study, wild type Bristol N2 and NL2099 (*rrf-3*(pk1426) II) strain were used. These strains were obtained from the *Caenorhabditis* Genetics Center (University of Minnesota).

### RNAi Clones and Selection of Phenotype

We have chosen *eri-1* and *lin-35* genes to explore the most suitable condition which gives enhanced RNAi sensitivity in wild type strain of *C. elegans*. The phenotype assayed were Unc (uncoordinated), Dpy (dumpy), Bli (blistering of cuticle) observed after inhibiting the expression of genes *unc -73*, *dpy – 9* and *bli-3* respectively.

### RNAi induced gene silencing

RNAi induced gene silencing was achieved using standard feeding protocol as described previously [5], [15]. In brief, NGM-IPTG agar plates were prepared by the addition of isopropyl isopropylthio-β-D-galactoside (IPTG; 5 Mm; Sigma, St. Louis, MI, Cat. No: I6758) and carbenicillin (25 mg/L; Sigma, St. Louis, MI, Cat. No: C138) to the freshly prepared NGM which was then poured onto 6 well culture plates (BD Falcon,Cat no. 353046) [16]. Separately, LB broth was prepared, autoclaved and added with 50 µg/ml ampicillin (Sigma, St. Louis, MI Cat. No: A0166) after cooling. The desired bacterial clone, expressing dsRNA for the target gene, was then inoculated into this sterile ‘LB-amp’ in a 2 ml microcentrifuge tube. The bacteria were grown for 6–8 hrs, at 37°C in a shaking incubator at 200 rpm. The 100 µl volume of actively growing culture bacteria expressing dsRNA was then seeded onto these 6-well NGM-IPTG-agar plates followed by an overnight incubation at 37°C for induction of double-stranded RNA synthesis by IPTG. Age synchronized embryos were added to these plates and worms were allowed to grow to early adulthood at 22°C for 48 hrs, for further studies. We used bacterial clones from the Ahringer RNAi library that was purchased from SA Biosciences (Cambridge, UK).

### Methods to induce enhanced gene silencing

Various procedures were employed towards enhancing the efficacy of RNAi induced gene silencing using the nematode model. Across each procedure, nematodes were fed on various dsRNA-synthesizing bacteria, as detailed in following subsections

#### Method I: RNAi clone of gene of interest co-incubated with RNAi clones for eri-1 and lin-35

In this method, age synchronized worms of N2 strain were grown for 48 hrs at 22°C onto NGM – IPTG plates seeded with bacteria expressing dsRNA for desired gene with following combinations of *eri-1* and *lin-35* gene.

- Incubation of worms with target RNAi clone/gene of interest
  In this treatment method, the embryos were grown on NGM plates with IPTG and carbenicillin, seeded with bacteria expressing dsRNA for gene of interest. After incubation of 48 hrs, worms were assayed for respective phenotypes.
- Target/gene of interest RNAi clone co-incubated with eri-1 RNAi clone
  Bacteria expressing dsRNA corresponding to the target gene and bacteria expressing dsRNA for eri-1, were mixed in equal volume (50 µl each from colonies with equal density) and seeded onto NGM plates with 5 mM IPTG and 25 mg/L carbenicillin followed by overnight incubation at 37°C. Synchronously obtained embryos were transferred to these plates and allowed to grow for 48 hrs before phenotypic characterization.
- Target/gene of interest RNAi clone co-incubated with lin-35 RNAi clone
  In this method, age synchronized worms were fed on bacteria producing dsRNA for both lin-35 and gene of interest. Before seeding, both dsRNA expressing bacterial cultures were mixed into equal volume by adding 50 µl from each culture. Post seeding, synchronously obtained embryos were transferred to these plates and allowed to grow for 48 hrs before phenotypic characterization.
- Target/gene of interest RNAi clone co-incubated with eri-1 and lin-35 RNAi clone
  In this method, dsRNA expressing clone of gene of interest was co-incubated with bacterial clones expressing dsRNA targeted for *eri-1* and *lin-35* genes. These bacterial clones were mixed in equal volume (33.3 µl each) before seeding onto assay plates for further analyses.

#### Method II: Pre-incubation with eri-1 RNAi clone followed by incubation under different conditions

In this treatment condition, age synchronized worms were first grown for 24 hrs at 22°C on NGM – IPTG plates seeded with bacteria expressing dsRNA for *eri-1*. After 24 hrs, worms were washed with M-9 buffer under sterile condition to remove any adhering bacteria then transferred onto new NGM-IPTG plates seeded with bacteria producing dsRNA of various combinations as detailed in following subsections. This method of pre-induction with *eri-1* RNAi clone, was performed under three different conditions to find out the most effective method.

- Pre-incubation with eri-1 RNAi clone followed by incubation with gene of interest RNAi clone
  In this method, age synchronized worms were first incubated with eri-1 RNAi clones. After 24 hrs of pre-incubation with eri-1, worms were further incubated for 24 hrs with bacteria expressing dsRNA for target gene. After 48 hrs post embryonic age, worms were analyzed for particular RNAi phenotype.
- Pre-incubation with eri-1 RNAi clone followed by co-incubation with gene of interest and eri-1 RNAi clones
  In this method, worms were initially pre-incubated at 22°C with bacteria expressing dsRNA for eri-1 gene for 24 hrs, and then it was followed by co-incubation with target gene and eri-1 RNAi clones together, for 24 hrs. Here, dsRNA expressing bacteria of target gene and eri-1 were mixed in equal volume (50 µl each) and seeded onto 6-well NGM-IPTG plates followed by overnight incubation at 37°C.
- Pre-incubation with eri-1 RNAi clone followed by co-incubation with gene of interest, eri-1 and lin-35 RNAi clones
  In this method, aged synchronized worms were added onto NGM –IPTG plates having dsRNA for *eri-1* gene for 24 hrs. After this, worms were washed using M-9 buffer and transferred onto plates seeded with RNAi bacteria expressing dsRNA corresponding to the target gene, *eri-1* gene and *lin-35* gene for 24 hrs. These RNAi clones were mixed in equal volume (33.3 µl of each RNAi clone) and seeded directly onto NGM – IPTG plates, followed by feeding of worms for 48 hours and further phenotypic analyses.

#### Method III: Pre-incubation with gene of interest RNAi clone followed by incubation under different conditions

In this method, worms were initially pre-incubated with RNAi clone of gene of interest for 24 hrs, followed by different co-incubations of RNAi enhancer clones with the incubation periods of 24 hrs. This method was performed under three different conditions:

- Pre-incubation with gene of interest RNAi clone followed by incubation with eri-1 RNAi clone
  In this method, worms were pre-incubated with RNAi clone of gene of interest, for 24 hours followed by incubation with bacteria producing dsRNA for eri-1. Further analysis of phenotype was performed after 48 hrs.
- Pre-incubation with gene of interest RNAi clone followed by co-incubation with gene of interest and eri-1 RNAi clones
  Worms were pre-incubated with RNAi clone of desired gene for 24 hrs and then co-incubated with eri-1 and gene of interest RNAi clones. These RNAi clones of eri-1 and gene of interest were mixed in equal volumes from colonies with same density.
- Pre-incubation with gene of interest RNAi clone followed by co-incubation with gene of interest, eri-1 and lin-35 RNAi clones
  Preincubation with gene of interest for 24 hours was followed by co-incubation in bacteria expressing dsRNA for three different genes – the gene of interest, *eri-1* and *lin-35*. The respective bacterial clones were mixed using equal volumes of each culture. Worms were assayed for phenotypes at 48 hours post embryonic age.

### Assay for analysis of phenotypic characterization

#### 1. Phenotypic analysis for Unc

In all the above mentioned methods, worms were analyzed using Leica S8AP0 stereozoom microscope for the uncoordinated phenotype, after treatment of 48 hrs. It was started with the scoring for the uncoordinated worms by comparing each RNAi treatment group to that of the control (N2-OP50) at a fixed time point. It can be performed either by prodding the worms with the help of poking lash or by tapping the worms on the head. Unc phenotype was counted for approximately 30-40 worms per group in triplicates and the percentage of worms showing phenotype in treated conditions was normalized against control values. The assays were repeated twice at two different days and the data for each group was statistically analyzed and plotted against control values. Unc phenotype was categorized into weak, moderate and strong phenotype.

*Weak phenotype*: Worms were able to move in a sinusoidal manner but could not progress forward or backward, movement being more responsive and energetic. It is indicated by a single asterisk *.

*Moderate phenotype*: Worms were unable to make complete sinusoidal movements but able to respond to mechanical stimulus, appeared slightly lethargic relative to untreated wild-type (N2) worms. It is presented by two asterisks * *.

*Strong phenotype*: Worms were unable to make complete sinusoidal movements and to respond to mechanical stimulus, appeared completely lethargic. It is depicted by three asterisks * * *.

#### 2. Phenotypic analysis for dumpy

RNAi dumpy phenotypes were analysed (N = 30–40 worms per plate) after 48 hrs of treatment at 22°C in all the above described methods using a Leica light microscope. Worms that were visibly shorter than wild type were scored as dumpy. The observation of this phenotype was classed as per three degrees of intensity viz weak, moderate and strong phenotype, depending upon the severity of the dumpiness. Assays were performed in triplicates and repeated twice and the data for each group was statistically analyzed and plotted against control values.

#### 3. Phenotypic analysis for blistering of cuticle

To observe the Bli phenotypes, 30–40 worms per group were analyzed using a Leica S8AP0 stereozoom microscope. Experiment was performed in triplicates at two different times. This phenotype was observed at a higher zoom, to visualize the blistering of cuticle in worms. Mutation in collagen genes and their biosynthetic pathway leads to abnormal morphology in *C. elegans* like blistering of cuticle. Blistering phenotype was also observed as weak, moderate and strong based on the intensity of effect.

#### Statistical analysis

All presented data were expressed as mean ± SEM. The statistical significance between two groups was determined using Student's *t*- test. P value of p<0.05 was considered as significant. Statistical analysis was done using Graph Pad Prism 5 software.

## Results

### Method I: RNAi clone of gene of interest co-incubated with RNAi enhancers (*eri-1* and *lin-35*) showed weaker RNAi phenotype

This method displayed comparatively weaker RNAi phenotype. The descriptions of the concerned methodologies applied, for respective phenotypes were as under.

#### Unc phenotype

**Figure 1:**
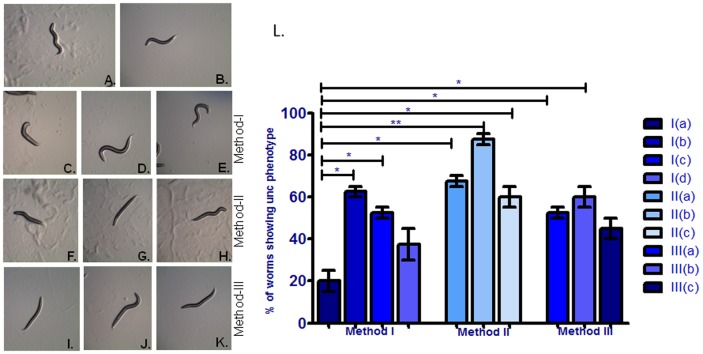
Effect of different employed RNAi methodology on uncoordinated phenotype in control N2 strain (A), RNAi induced gene silencing of *unc-73* gene (B), *unc-73* RNAi clone co-incubated with *eri-1* RNAi clone (C), *unc-73* RNAi clone co-incubated with *lin-35* RNAi clone (D) *unc-73* RNAi clone co-incubated with *eri-1* and *lin-35* RNAi clones (E), Pre-incubation with *eri-1* RNAi clone followed by incubation with *unc-73* RNAi clone (F) Pre-incubation with *eri-1* RNAi clone followed by co-incubation with *unc-73* and *eri-1* RNAi clones(G),Pre-incubation with *eri-1* RNAi clone followed by co-incubation with *unc-73*, *eri-1* and *lin-35* RNAi clones(H), Pre-incubation with *unc-73* RNAi clone followed by incubation with *eri-1* RNAi clone (I) Pre-incubation with *unc-73* RNAi clone followed by co-incubation with *unc-73* and *eri-1* RNAi clones (J) Pre-incubation with *unc-73* RNAi clone followed by co-incubation with *unc-73*, *eri-1* and *lin-35* RNAi clones (K). Fig 1L is a graphical presentation of worms showing percentages of unc phenotypes examined by three different RNAi methodologies.*p<0.05, **p<0.01, NS - Not significant.

Under control conditions, worms move in a sinusoidal manner whereas the worms exhibiting reduced expression of *unc-73* either via knockout mutation or in case of RNAi induced gene silencing, exhibit an uncoordinated movement pattern thereby not showing the typical sinusoidal wave motion as wild type worms do. Unc phenotypes arise due to defects in the development or function of the neuromuscular system. When wild-type *C. elegans* were fed on bacteria producing *unc-73* dsRNA, most of the worms were unaffected by dsRNA to this neural development gene, only 20% of worms displayed weak unc phenotype (Fig. 1B,1L) Whereas this phenotype was significantly (p<0.05) enhanced in wild type worms when co-incubated with both *unc-73* and *eri-1* dsRNA (Fig. 1C,1L). In this treatment condition, 3.1 fold increase in RNAi efficiency was observed as compared to worms fed on bacteria expressing only *unc-73* dsRNA. Although co-incubation with *unc-73* and *lin-35* genes exhibited weaker unc phenotype as compared to co-incubation with *unc-73* and *eri-1* RNAi clone but the percentage of worms, showing unc phenotype was 2.6 fold higher than worms fed only with *unc-73* dsRNA so it demonstrated that *lin-35* also increased RNAi efficiency to dsRNA (Fig. 1D,1L). To further examine the effects of both RNAi enhancers together we grown worms on bacteria producing *unc-73*, *eri-1* and *lin-35* dsRNA. In this treatment condition we didn’t observe significant enhancement in RNAi sensitivity to dsRNA (Fig 1E, 1L, and Table 1).

**Table 1:** Employed three different RNAi methodologies in wild type N2 strain of *C. elegans* for uncoordinated phenotype.

RNAi experiment	Extent of phenotype	% of worms showing phenotype (Mean ± SEM)
**Method I**		
N2 - Control	WT	WT
unc-73	+	20.00±5.00
unc-73+ eri-1	+ +	62.50±2.50
unc-73+lin35	+	52.50±2.50
unc-73+ eri-1+lin35	+	35.50±7.50
**Method II**		
unc-73	+ +	67.50±2.50
unc-73+ eri-1	+ + +	87.50±2.50
unc-73+ eri-1+lin35	+	60.00±5.00
**Method III**		
eri-1	+ +	52.50±2.50
unc-73+ eri-1	+ +	60.00±5.00
unc-73+ eri-1+lin35	+ +	45.00±5.00

Method I, RNAi clone of *unc-73* co-incuabted with RNAi enhancers (*eri-1* and *lin-35*); Method II, Pre-incubation with *eri-1* RNAi clone followed by incubation with *unc-73* RNAi clone, co-incubation with *eri-1* and *unc-73* RNAi clones and co-incubation with *unc-73*, *eri-1* and *lin-35* RNAi clones; Method III, Pre-incubation with *unc-73* RNAi clone followed by incubation with *eri-1*, co-incubation with *unc-73* and *eri-1* RNAi clones and co-incubation with *unc-73*, *eri-1* and *lin-35* RNAi clones. unc, uncoordinated; WT, wild-type. +,weak phenotype; ++, medium phenotype; +++, strong phenotype.

#### Dumpy phenotype

**Figure 2:**
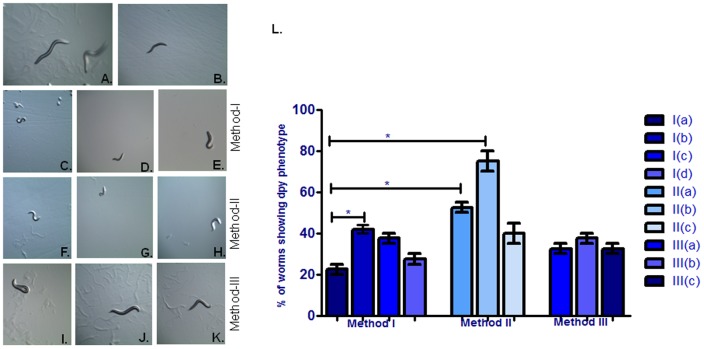
Effect of different employed RNAi methodology on dumpy phenotype in control N2 strain (A), RNAi induced gene silencing of *dpy-9* gene (B), *dpy-9* RNAi clone co-incubated with *eri-1* RNAi clone (C), *dpy-9* RNAi clone co-incubated with *lin-35* RNAi clone (D) *dpy-9* RNAi clone co-incubated with *eri-1* and *lin-35* RNAi clones (E), Pre-incubation with *eri-1* RNAi clone followed by incubation with *dpy-9* RNAi clone (F) Pre-incubation with *eri-1* RNAi clone followed by co-incubation with *dpy-9* and *eri-1* RNAi clones(G),Pre-incubation with *eri-1* RNAi clone followed by co-incubation with *dpy-9*, *eri-1* and *lin-35* RNAi clones(H), Pre-incubation with *dpy-9* RNAi clone followed by incubation with *eri-1* RNAi clone (I) Pre-incubation with *dpy-9* RNAi clone followed by co-incubation with *dpy-9* and *eri-1* RNAi clones (J) Pre-incubation with *dpy-9* RNAi clone followed by co-incubation with *dpy-9*, *eri-1* and *lin-35* RNAi clones (K). Fig 1L is a graphical presentation of worms showing percentages of dumpy phenotypes examined by three different RNAi methodologies. *p<0.05, NS - Not significant.

Knockdown of *dpy-9* by RNAi in wild type worms displayed post-embryonic weak dumpy phenotypes. However, when worms were raised on bacteria that co-expressed dsRNA of both *dpy-9* and *eri-1*, the proportion of dpy worms was significantly (p<0.05) enhanced (fig. 2C, 2L) by 1.87 folds, as compared to worms raised on *dpy-9* RNAi clones. Whereas, co-incubation with *dpy-9* and *lin-35* as well as co-incubation with *dpy-9*, *eri-1* and *lin-35* RNAi clones didn’t exhibit significant increase in RNAi phenotype (Fig. 2D, 2E, 2L and Table 2).

**Table 2:** Employed three different RNAi methodologies in wild type N2 strain of *C.elegans* for dumpy phenotype.

RNAi experiment	Extent of phenotype	% of worms showing phenotype (Mean ± SEM)
**Method I**		
N2 – control	WT	WT
dpy-9	+	22.50±2.50
dpy-9+ eri-1	+ +	42.50±2.50
dpy-9+lin35	+	37.50±2.50
dpy-9+ eri-1+lin35	+ +	27.50±2.50
**Method II**		
dpy-9	+ +	52.50±2.50
dpy-9+ eri-1	+ + +	75.00±5.00
dpy-9+ eri-1+lin35	+	40.00±5.00
**Method III**		
eri-1	+	32.50±2.50
dpy-9+ eri-1	+	37.50±2.50
dpy-9+ eri-1+lin35	+	32.50±2.50

Method I, RNAi clone of *dpy-9* co-incuabted with RNAi enhancers (*eri-1* and *lin-35*); Method II, Pre-incubation with *eri-1* RNAi clone followed by incubation with *dpy-9* RNAi clone, co-incubation with *eri-1* and *dpy-9* RNAi clones and co-incubation with *dpy-9*, *eri-1* and *lin-35* RNAi clones; Method III, Pre-incubation with *dpy-9* RNAi clone followed by incubation with *eri-1*, co-incubation with *dpy-9* and *eri-1* RNAi clones and co-incubation with *dpy-9*, *eri-1* and *lin-35* RNAi clones. dpy, dumpy; WT, wild-type. +,weak phenotype; ++, medium phenotype; +++, strong phenotype.

#### Blistered Phenotype

Although, RNA interference (RNAi) targeted against *bli-3* results in complex phenotypic defects including abnormal cuticle morphology and blistered phenotype. However, in our study, only 22.5% worms showed weak blistered phenotype when they were fed onto *bli-3* RNAi bacteria. On the other side, knock down of *bli-3* and *eri-1* together using feeding RNAi with bacteria did not show any better blistered phenotype with the efficiency of 32.5%. Besides, when bacteria that expressed dsRNA of *bli-3+ lin-35* and *bli-3+eri-1+lin-35* were mixed together and fed to worms, the proportion of Bli worms and cuticle morphology was similar to worms fed only *bli-3* RNAi bacteria. In this phenotype, *eri-1* and *lin-35* did not enhance RNAi sensitivity in wild type worms.

### Method II Pre-induction with *eri-1* followed by gene of interest and *eri-1* showed strongest RNAi phenotype

In this method, worms were first fed for 24 hrs on bacteria expressing *eri-1* dsRNA and then transferred onto desired gene, desired gene + *eri-1* and desired gene + *eri-1* +*lin-35* RNAi bacteria. Phenotypic characterization of individual gene was performed as previously described for uncoordinated, dumpy and for blistered phenotype.

#### Unc phenotype

After transferring worms from *eri-1* seeded RNAi bacteria to *unc-73* seeded RNAi food, we observed significantly (p<0.05) 3.4 fold increase in RNAi efficiency as compared to worms fed only *unc-73* dsRNA (Fig. 1F,1L and Table 1). Similarly, when worms were first pre-incubated with *eri-1* RNAi food and after that co-incubated with *unc-73* and *eri-1* seeded RNAi bacteria, RNAi phenotype was significantly (p<0.01) 4.4 folds higher as compared *unc-73* RNAi clones (Method 1(a) (Fig 1G,1L). In this method, worms showed strongest RNAi phenotype with highest RNAi sensitivity to dsRNA. Percentage of worms showing this phenotype was 87.50±2.50. These results suggest that this method provides robust and most efficient gene silencing in wild type worms. In contrast, RNAi efficiency was reduced in worms pre-incubated with *eri-1* followed by *unc-73* + *eri-1* + *lin-35* RNAi food in comparison to *eri-1* to gene and gene + *eri-1* method and worms showed weak unc phenotype but this combination also showed 1.6 fold increase in RNAi efficiency as compared to method I i.e. without pre-incubation of *eri-1*, worms were cultured onto mixture of *unc-73* + *eri-1* +*lin-35* dsRNA.

#### Dumpy phenotype

When *eri-1* silenced worms were raised on bacteria producing *dpy-9* dsRNA then worms showed significantly (p<0.05) enhanced RNAi phenotype as compared to *dpy-9* only (method Ia).In this method, worms showed medium dumpy phenotype. Whereas, worms exhibited more severe dumpy phenotype when they were transferred from *eri-1* RNAi food to *eri-1* +*dpy-9* RNAi food with 3.3 fold increase in the RNAi efficiency in wild type background when compared to without prior induction with *eri-1*, grown onto *dpy-9* RNAi clone (Fig. 2G, 2L and Table 2). By using this method, RNAi efficiency was significantly (p<0.05) increased. However, pre-incubated worms with *eri-1* dsRNA were fed onto bacteria expressing mixture of *dpy-9* +*eri-1*+ *lin-35* dsRNA showed weak dumpy phenotype.

#### Blistered Phenotype

**Figure 3:**
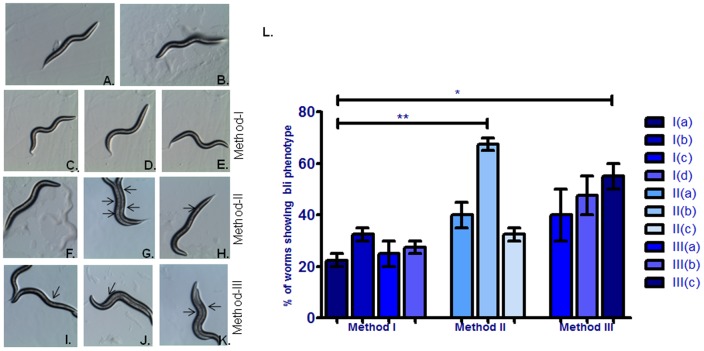
Effect of different employed RNAi methodology on blistering of cuticle phenotype in control N2 strain (A), RNAi induced gene silencing of *bli-3* gene (B), *bli-3* RNAi clone co-incubated with *eri-1* RNAi clone (C), *bli-3* RNAi clone co-incubated with *lin-35* RNAi clone (D) *bli-3* RNAi clone co-incubated with *eri-1* and *lin-35* RNAi clones (E), Pre-incubation with *eri-1* RNAi clone followed by incubation with *bli-3* RNAi clone (F) Pre-incubation with *eri-1* RNAi clone followed by co-incubation with *bli-3* and *eri-1* RNAi clones(G),Pre-incubation with *eri-1* RNAi clone followed by co-incubation with *bli-3*, *eri-1* and *lin-35* RNAi clones(H), Pre-incubation with *bli-3* RNAi clone followed by incubation with *eri-1* RNAi clone (I) Pre-incubation with *bli-3* RNAi clone followed by co-incubation with *bli-3* and *eri-1* RNAi clones (J) Pre-incubation with *bli-3* RNAi clone followed by co-incubation with *bli-3*, *eri-1* and *lin-35* RNAi clones (K). Fig 3L is a graphical presentation of worms showing percentages of bli phenotypes examined by three different RNAi methodologies.*p<0.05, NS - Not significant.

When wild-type *C. elegans* were fed on bacteria producing only *bli-3* dsRNA and co-incubation with *bli-3*, *eri-1*, *lin-35* dsRNA, after pre-incubation with *eri-1*, didn’t exhibited significant enhancement in RNAi efficiency. In these conditions, worms displayed weak blistered- phenotype. This RNAi efficiency was significantly (p<0.05) 3.3 folds enhanced when worms were co-incubated with *bli-3* and *eri-1* RNAi clones after pre-incubation with *eri-1* when compared to worms fed onto *bli-3* RNAi food (method Ia). In this group, most of the worms displayed more abnormal cuticle morphology as compared to other groups (Fig 3G, 3L Table 3).

**Table 3:** Employed three different RNAi methodologies in wild type N2 strain of *C.elegans* for blistering of cuticle phenotype.

RNAi experiment	Extent of phenotype	% of worms showing phenotype (Mean ± SEM)
**Method I**		
N2 – control	WT	WT
bli-3	+	22.50±2.50
bli-3+ eri-1	+	32.50±2.50
bli-3+lin35	+	25.00±5.00
bli-3+ eri-1+lin35	+	27.50±2.50
**Method II**		
bli-3	+	40.00±5.00
bli-3+ eri-1	+ + +	67.50±2.50
bli-3+ eri-1+lin35	+	32.50±2.50
**Method III**		
eri-1	+	40.00±10.00
bli-3+ eri-1	+ +	47.50±7.50
bli-3+ eri-1+lin35	+	55.00±5.00

Method I, RNAi clone of *bli-3* co-incuabted with RNAi enhancers (*eri-1* and *lin-35*); Method II, Pre-incubation with *eri-1* RNAi clone followed by incubation with *bli-3* RNAi clone, co-incubation with *eri-1* and *bli-3* RNAi clones and co-incubation with *bli-3*, *eri-1* and *lin-35* RNAi clones; Method III, Pre-incubation with *bli-3* RNAi clone followed by incubation with *eri-1*, co-incubation with *bli-3* and *eri-1* RNAi clones and co-incubation with *bli-3*, *eri-1* and *lin-35* RNAi clones. bli, Blistering of cuticle; WT, wild-type. +, weak phenotype; ++, medium phenotype; +++, strong phenotype.

### Method III: Pre – induction with gene of interest followed by *eri-1* showed average RNAi phenotype

#### Unc phenotype

When *unc-73* silenced worms transferred onto *eri-1* seeded dsRNA, worms showed significantly 2.6 folds (p<0.05) enhanced RNAi phenotype when compared to worms which have grown onto bacteria which expressed *unc-73* dsRNA (method Ia). This method showed similar RNAi efficiency as observed in method IIa in which worms were first pre-incubated with *eri-1* dsRNA then onto *unc-73* RNAi food. Similarly, pre-induction with unc-73 RNAi clones followed by co-incubation with *unc-73, eri-1* RNAi clones also showed 3 folds (p<0.05) increased RNAi efficiency when compared to *unc-73* RNAi clones or method 1a group. On the other side, third combination of this method i.e. pre-incubation with *unc-73* RNAi clone followed by co-incubation with *unc-73*+*eri-1*+*lin-35* RNAi clones didn’t significantly affect RNAi efficiency when compared to method Ia.

#### Dumpy phenotype

We did not observe enhanced RNAi efficiency in this phenotype using this method. Although worms showed similar phenotype as observed in method I when incubated with bacteria expressing *dpy-9* dsRNA. In all groups, worms showed weak dumpy phenotype.

#### Blistered Phenotype

Pre-induced worms with *bli-3*, when co-incubated with bacteria producing dsRNA of *eri-1* and *bli-3* + *eri-1*didn’t exhibit significant increases in RNAi efficiency but this efficiency of RNAi was significantly (p<0.05) 2.4 folds enhanced when these *bli-3* pre-incubated worms grown onto *bli-3*+*eri-1*+*lin-35* when compared to worms grown onto *bli-3* RNAi food (method Ia).

### Pre-induced worms with *eri-1* followed by gene + *eri-1* mixture exhibited stronger RNAi phenotype than *rrf-3* mutant

**Figure 4:**
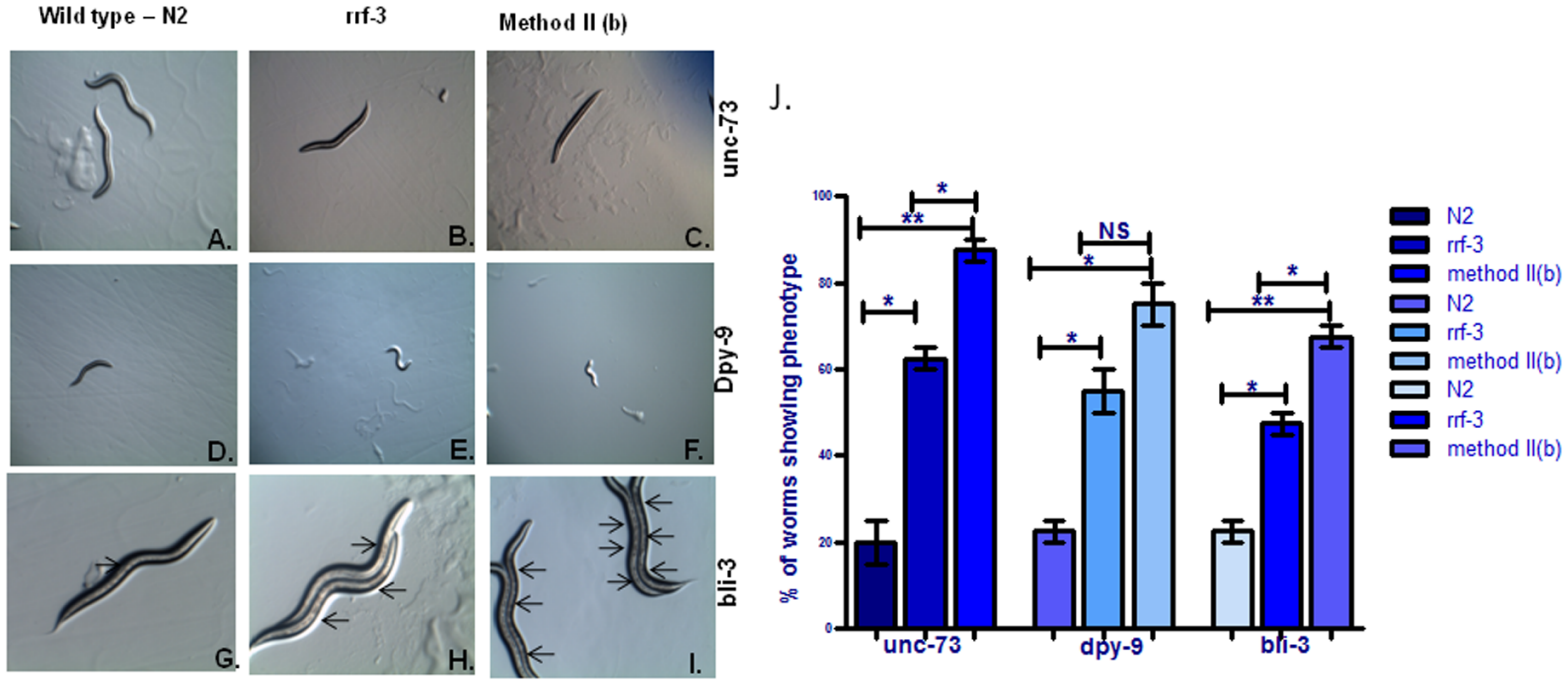
Comparison of newly identified RNAi methodology performed in RNAi induced gene silencing of *unc-73* in wild type background (A), RNAi induced gene silencing of *unc-73* in *rrf-3* background (B),Pre-incubation with *eri-1* RNAi clone followed by co-incubation with *unc-73* and *eri-1* RNAi clones in wild type background (C), RNAi induced gene silencing of *dpy-9* in wild type background (D), RNAi induced gene silencing of *dpy-9* in *rrf-3* background (E),Pre-incubation with *eri-1* RNAi clone followed by co-incubation with *dpy-9* and *eri-1* RNAi clones in wild type background (F), RNAi induced gene silencing of *bli-3* in wild type background (G), RNAi induced gene silencing of *bli-3* in *rrf-3* background (H),Pre-incubation with *eri-1* RNAi clone followed by co-incubation with *bli-3* and *eri-1* RNAi clones in wild type background (I). Fig 4J is a graphical presentation of worms showing percentages of unc/dpy/bli phenotypes examined by different RNAi methodologies. *p<0.05,**p<0.01, NS - Not significant.

In our studies, method IIb (*eri-1* to gene +*eri-1*) showed highest RNA sensitivity in all the phenotypes as described previously. We further wanted to check whether this method shows similar or enhanced RNAi efficiency as using *rrf-3* mutant so we compared this phenotype using *rrf-3* mutant which have already been reported to enhance RNAi efficiency in number of studies. When we compared unc phenotype, we observed 1.4 fold increase in RNAi efficiency in worms pre-incubated with *eri-1* then co-incubated with *unc-73* +*eri-1* RNAi food (method IIb) (Fig. 4C,4J) as compared to worm grown onto *rrf-3* mutant background (p<0.05) (Fig. 4B,4J). In this condition (method IIb), worms showed strongest unc phenotype.On the other side, in N2 strain, RNAi of *unc-73* exhibited similar sinusoidal movement phenotype as in wild type condition. (Table 4). However, using dpy phenotype, we did not found significant increase in RNAi efficacy using method IIb when compared to *rrf-3* mutant strain (Fig. 4E Vs 4F). In this phenotype, we found almost similar RNAi efficiency and in this phenotype, method IIb gives similar RNAi phenotype as observed using *rrf-3* mutant. However, in bli phenotype, worms showed more abnormal cuticle morphology employing method IIb (Fig. 4I) than to *rrf-3* (Fig. 4H) and 1.4 fold increase in efficiency of RNAi was observed (p<0.05) (Fig. 4J Table 4).

**Table 4:** Comparison of RNAi methodology performed in wild type backgroud, *rrf-3* background and carried out using identified method II (b), pre-incubation with *eri-1* RNAi clone followed by co-incubation with gene of interest and *eri-1* RNAi clones.

RNAi	N2 phenotype*	RRF-3 phenotype*	N2: eri-1 to gene + eri-1 phenotype*
unc-73	WT	unc + +	unc + + +
dpy-9	Dpy +	Dpy + + +	Dpy + + +
bli-3	Bli +	Bli+ +	Bli+ + +

WT, wild-type; unc, uncoordinated; dpy, dumpy; bli, Blistering of cuticle; +, weak phenotype; ++, medium phenotype; +++, strong phenotype.

## Discussion

Here we describe a method of RNAi induced gene silencing wherein efficiency of gene silencing is enhanced via reducing levels of factors that interfere with the dsRNA or its products during the silencing process. We used *unc-73* gene to produce uncoordinated phenotype. Silencing of *unc-73* is already known for its post embryonic uncoordinated phenotypes in number of studies [8], [15], [17]–[19]. In *C. elegans*, *unc-73* (F55C7.7) is predicted to encode a guanine nucleotide exchange factor (GEF), a regulator of Rac GTPase which is expressed in neurons, muscles and in the gonad [20], [21] and plays an important role in axon guidance [22]. Previously, it has been shown that the *unc-73* (e936) allele has a G-to-U mutation at the first nucleotide of intron which leads to an uncoordinated phenotype [22]. Another phenotype used in this study, was dumpy, caused as a result of silencing of *dpy-9* (T21D12.2) encoding a cuticular collagen family member. Silencing of this gene is related to cuticle dysfunction and defects in epithelium which results in dumpy phenotype [8]. It has been shown that Glycine substitutions within the Gly-X-Y portion of the collagens can cause the morphological dumpy (Dpy) phenotypes [23]. Besides, Blistering of cuticle was a third selected phenotype which was used in the present study. Knock-down of *bli-3* using feeding RNAi with bacteria results in a blistered phenotype, have also been shown previously [8]. This gene encodes a large homolog of dual oxidase (‘Ce-Duox1’) which is required for dityrosine cross-linking of collagen, and thus for cuticular integrity [24]. This gene plays a key role in collagen/cuticle biosynthesis.

In our studies, the absence of *unc-73*, *dpy-9* and *bli-3* activity led to weak phenotype in wild type background. Previous studies have postulated that the nervous system of *C. elegans* is partially refractory to RNAi [25] and in wild type *C. elegans* (N2 Bristol), high throughput RNAi screens might reveal partial susceptibility of certain genes to dsRNA induced inhibition [8]. Several genetic backgrounds have been described in which animals are more sensitive to RNAi. In the present study, we tested three of these backgrounds, *eri-1, lin-15* and *rrf-3*. In addition, it has been revealed that the consistency and reproducibility of high throughput RNAi screens could be improved using *eri-1* (enhanced RNA interference), a conserved antagonist of RNAi which is preferentially expressed in neurons and the somatic gonad. It has been shown that the absence of *eri-1* activity led to an obvious enhancement in the sensitivity to RNAi, in a range of target genes, including neuronal genes [9]. The *eri-1* is an RNaseT enzyme which belongs to the DEMDh exonuclease family with a SAP domain and a DEDDh-like 3′→5′ exonuclease domain that can degrade double-stranded RNA with 3′ overhangs. It was initially found in the nematode, *C. elegans*, and this ribonuclease has since been found to have orthologs in a broad range of genomes. In previous study, it has also been demonstrated that in mouse, deletion of m*eri-1* which is orthologue of *eri-1*, by RNAi can increase the sensitivity of RNAi [26] Similarly, in humans, 3′hExo is *eri-1* orthologue, plays a important role in histone mRNA biogenesis and metabolism [27], elimination of its activity have been shown to increase the efficacy of RNAi in these systems. In addition, in the fission yeast *S. pombe*, loss of *eri-1* activity caused increased levels of small interfering RNAs (siRNAs) corresponding to centromeric repeats and displayed high sensitivity of RNAi [28]. In our study, we also observed enhanced efficiency of RNAi when worms were grown onto mixture of *eri-1* and gene of interest producing RNAi bacteria but this efficiency was further significantly enhanced in worms when pre-induced with *eri-1* then transferred onto *eri-1* and gene of interest expressing RNAi bacteria. It demonstrated that worms when fed onto bacteria expressing *eri-1* dsRNA, accumulate more siRNAs than do wild-type animals, suggesting that *ERI-1* plays a regulatory role in the RNAi machinery, in which short interfering double stranded RNA pairs up with complementary strands of messenger RNA, marking them for degradation.

Second RNAi enhancer used in this study was *lin-35*. This gene encodes the worm tumour suppressor gene *p105Rb* or retinoblastoma protein (Rb) ortholog which is linked to synMuv B family of chromatin modifying genes. Although, recent studies have revealed that *C. elegans* strain having mutations in *lin-35* showed enhanced embryonic and post embryonic RNAi responses, including neuronal RNAi responses [10], [11] but we found slight enhancement in RNAi efficiency when worms were grown onto mixture of *lin-35* and target gene expressing RNAi food. This effect may be due to the varied sensitivity of RNAi because the effectiveness of RNAi also varies from gene to gene.

After analyzing the enhanced RNAi phenotypes, using *eri-1* and *lin-35* gene knockdowns, the phenotypes were further assessed using the two enhancers together with the target gene, but no such elevation in RNAi efficiency was observed. This effect probably is associated with dilution effect of RNAi bacteria which produces dsRNA, reducing the manifestation of the corresponding phenotype. However, mixture of target gene and *eri-1* also showed dilution effect but another probable reason for enhanced RNAi phenotype might be the effect of *eri-1* gene. On silencing two corresponding genes together (method II) despite the possible dilution effect, marked enhancement in RNAi efficiency is observed. However, no such enhancement is observed on silencing the target gene along with *eri-1* and *lin-35*, may be because of much higher dilution effect masking the RNAi efficiency.

When we observed enhanced efficiency of RNAi in all the phenotypes using method II (pre incubation in *eri-1* RNAi clone followed by co-incubation in *eri-1* and gene-of-interest RNAi clines) then to validate the obtained results using pre-incubation with *eri-1*, we compared the RNAi phenotypes using *rrf-3* mutant. We found stronger RNAi phenotypes by using these methods in unc and bli phenotype as compared to *rrf-3* animal and almost equivalent in dpy phenotype. In *C. elegans*, there are four putative RNA-directed RNA polymerases [RdRP] gene including *ego-1, rrf-1, rrf-2* and *rrf-3*. Among the all four RdRP genes, *rrf-3* deletion mutant has been reported to enhance sensitivity to RNAi including neuronal genes [6], [29]. Although, in our studies *rrf-3* mutant provided enhanced RNAi phenotype as compared to wild type background but similar or reduced phenotype, when compared with method II. The enhanced efficiency in the described method could be as a result of sufficient knocking down of the RNAse T effect before the incubation of the worms in RNAi clones targeted for the gene of interest. The absence of RNAse T effect helps in potential stability of the dsRNA and related products in the process of RNA interference. Further, co-incubation of the gene-of-interest RNAi clone with that of *eri-1* ensures the reduced RNAse T activity through the process hence enhancing the efficacy of RNAi.
